# Glioblastoma Following Traumatic Brain Injury: Case Report and Literature Review

**DOI:** 10.7759/cureus.8019

**Published:** 2020-05-07

**Authors:** Raimondas Juškys, Žilvinas Chomanskis

**Affiliations:** 1 Department of Anatomy, Histology and Anthropology, Faculty of Medicine, Vilnius University, Vilnius, LTU; 2 Department of Neurology and Neurosurgery, Faculty of Medicine, Vilnius University, Vilnius, LTU

**Keywords:** traumatic brain injury, intracerebral hemorrhage, glioblastoma, case report

## Abstract

The association between traumatic brain injury and brain cancer is a matter of debate. The available literature is sparse and yields conflicting results. Even though there is a pathophysiological rationale for post-traumatic intracranial cancerogenesis, the direct link still has not been proven. Here we present a case of a patient who developed glioblastoma multiforme four years following the traumatic intracerebral hemorrhage. In addition, we provide a brief review of the relevant literature.

## Introduction

The causal role between traumatic brain injury (TBI) and development of malignant brain tumor remains a matter of debate. There is a limited amount of literature exploring this topic. Theoretical and experimental data support such association, but currently available epidemiological studies yield conflicting results. Here we present a case of a male patient who developed glioblastoma (GBM) at the same location as did the former traumatic intracerebral hemorrhage (ICH) occurred four years ago. In addition, we have briefly reviewed available literature with an emphasis on relevant epidemiological studies and potential pathophysiological mechanisms that explain the link between trauma and gliomagenesis.

## Case presentation

**Figure 1:**
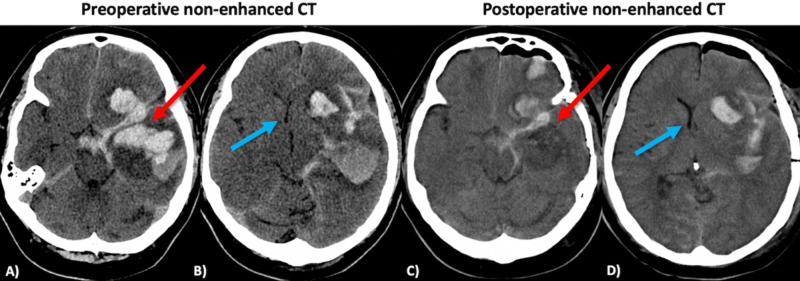
Preoperative (A,B) and postoperative (C,D) non-enhanced CT scans. A traumatic intracerebral hematoma with perifocal edema and subarachnoid hemorrhage extending into Sylvian fissure and basal cisterns was found upon the initial imaging (red arrow, slice A). The midline shift was equal to 9 mm (blue arrow, slice B). As a major part of hematoma in the temporal lobe was evacuated (red arrow, slice C), the midline shift diminished from 9 to 4 mm (blue arrow, slice D).

Our patient was a 47-year-old male who suffered a moderate TBI as a result of an accident of falling down the stairs in 2014. He was brought to the emergency department at a local university hospital with a Glasgow Coma Scale (GCS) score of 12. An initial neurological examination revealed a moderate aphasia, right-sided hemiparesis, and a positive Babinski sign on the right. Urgent head CT showed a left-sided frontotemporal ICH with a midline shift of 9 mm (Figures 1A, 1B). The patient underwent an emergency pterional craniotomy and hematoma evacuation. There was no evidence of tumor during the intraoperative period. Next day, postoperative CT scan showed a diminished midline shift to 4 mm and the remnants of hematoma (Figures 1C, 1D). The postoperative period was uneventful. The patient gradually improved and was discharged for further rehabilitation after 12 days with a GCS score of 15, mild motor aphasia, and slight right-sided hemiparesis. The patient showed up for the follow-up after two years in 2016. The clinical condition was satisfactory, no focal neurological signs were observed, and the patient complained only of easy fatigability and mild intermittent head pains in the region of craniotomy. No need for an additional neuroimaging was indicated at that moment as the patient did not show any signs of neurological deficits.

**Figure 2:**
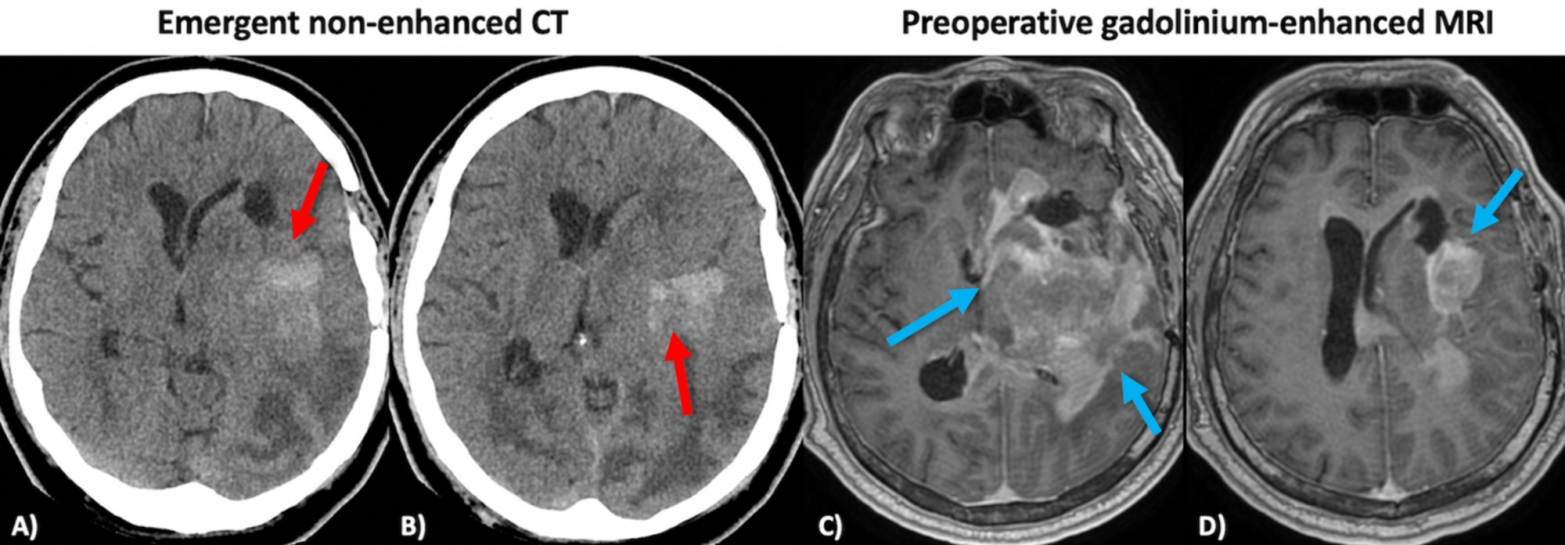
Emergent non-enhanced CT scan four years after the primary TBI (A,B) and gadolinium-enhanced MRI scan before stereotactic brain biopsy (C,D). Emergent non-enhanced CT revealed an atypically appearing spontaneous ICH in the area of traumatic scar, the same location where traumatic ICH has occurred back in 2014 (red arrows, slices A,B). Consequent gadolinium-enhanced MRI showed a heterogeneous, contrast-enhancing, and diffusely infiltrating mass (blue arrows, slices C,D). Glioblastoma, IDH negative was confirmed pathologically after the stereotactic brain biopsy. The GBM is centered in the same area where previous traumatic ICH occurred four years ago. ICH, intracerebral hemorrhage; IDH, isocitrate dehydrogenase; TBI, traumatic brain injury.

In 2018, the patient was brought to the emergency department at the same institution due to a sudden onset of severe headache, right-sided weakness, and altered mental status. The patient had a GCS score of 12. Neurological examination was significant for severe aphasia, right-sided hemiplegia, and a positive Babinski sign on the right. An urgent CT scan was performed, revealing a vague spontaneous ICH in the left temporoparietal region (Figures 2A, 2B). Due to atypical radiological appearance, the patient underwent a contrast-enhanced MRI scan to evaluate a potential secondary cause of the hemorrhage. A heterogeneous, contrast-enhancing frontotemporoparietal mass with extension to basal ganglia and corpus callosum was identified (Figures 2C, 2D). Next day, the patient underwent a stereotactic brain biopsy of the lesion. Histopathological evaluation was consistent with the diagnosis of GBM, isocitrate dehydrogenase (IDH) negative. Due to the advanced state of cancer, a radical surgical intervention was not an option. The postoperative period was uneventful, and the patient was discharged home to recover before an adjuvant radiochemotherapy course. Unfortunately, the patient's condition continued to deteriorate, and he passed away three weeks following the biopsy.

## Discussion

Despite the limited literature, there is some evidence suggesting the association between TBI and cerebral tumorigenesis. Several epidemiological studies examined the trauma-malignancy interrelationship, yielding conflicting results. Some smaller reports found an increased risk of neoplasia among patients with a history of TBI, whereas larger-scale epidemiological studies failed to prove a significantly elevated risk following the head injury.

Population-based Taiwanese cohort involving 5,007 patients with prior TBI found 4.67 times greater chance to develop intracranial malignancy within three years post-injury as compared with a control group. In addition, the authors reported a statistically significant association between TBI severity and the risk of post-traumatic malignancy [1]. Another case-control study examined 231 patients with a primary brain tumor and identified an association between former TBI and consequent cerebral neoplasm (odds ratio, 1.49). Besides, investigators found that two or more TBIs resulted in 3.14 times higher risk of brain cancer, suggesting frequency-hazard correlation [2]. Similarly, a multicentric case-control study involving 540 children with malignant brain tumor found that a history of head injury increased the probability of malignancy by 40%, and if there was an associated loss of consciousness, the risk increased up to 60%. Interestingly, the authors also concluded that a combination of complicated childbirth (either birth trauma or forceps delivery) and head injury later in life was associated with 2.6 times greater risk, suggesting an accumulative effect of repetitive traumatic insults [3].

On the other hand, the Swedish cohort study analyzing more than 311,000 patients found no significant association between TBI and the risk of primary brain tumor formation [4]. Similarly designed Danish study involving over 228,000 participants showed a minor, statistically insignificantly increased incidence of brain tumors formation among those with prior head injury [5]. Both studies did not take into account the severity and/or the number of traumatic insults experienced by the patients, potentially ignoring the key factors that are likely involved in post-traumatic tumor formation. Another recent population-based Danish cohort of more than 400,000 participants again found no correlation between structural brain damage and risk of cerebral cancerogenesis during five years of follow-up. However, this study involved not only TBIs, but also ischemic and hemorrhagic strokes, possibly tangling post-traumatic risk-related findings [6].

Even though epidemiological data remain inconclusive concerning the link between TBI and intracranial malignancy, there is some evidence that traumatic injury might considerably contribute to the gliomagenesis. Several rodent experimental studies showed that intracerebral trauma enhances the rate of glial tumors formation in rats already exposed to a potent cancerogen, suggesting that traumatic injury might act as a contributing factor in a context of genetic and/or environmental predisposition [7,8].

There are few hypotheses explaining post-traumatic glioma pathogenetic mechanisms, namely inflammatory response and neural stem cells migration/differentiation at the site of the primary injury. Inflammation is a physiological response to a tissue damage throughout all bodily tissues, including the brain. Minutes following the trauma, mobilization of resident microglia and consequent chemoattraction of peripheral immune cells take place, which play a key role in the inflammatory response. Somewhat surprisingly, persistent state of chronic neuroinflammation may last for many years after a single TBI, explaining why such a time-demanding process as oncogenesis might evolve in the first place [9]. This inflammatory microenvironment contributes to the neoplastic proliferation by two main interrelated mechanisms: formation of reactive oxygen species (ROS) is thought to induce DNA damage and genetic instability, whereas release of various immunomodulatory molecules (namely cytokines, prostaglandins, and chemokines) activates oncogenic transcription factors. Both processes eventually lead to oncogene activation and tumor suppressor gene inactivation, promoting gliomagenesis [10,11]. In addition to the immune response, there is evidence that trauma stimulates glial proliferation that becomes uncontrolled in the setting of different oncogenic growth factors and ROS-related oxidative stress, eventually contributing to malignant neoplasm formation [12]. Similarly, since the injury facilitates migration and mitotic activity of neural stem and progenitor cells, DNA damage with genetic alterations during this sensitive time period has been experimentally shown to induce GBM growth [13,14]. In fact, even terminally differentiated cells like astrocytes and neurons are capable to give rise to GBM in the context of the injury-altered environment by the process of dedifferentiation [15]. Undoubtedly, there are more mechanisms involved in the complex process of post-traumatic gliomagenesis, but only further experimental studies will reveal precise pathophysiological cascades that take place following the injury.

## Conclusions

Post-traumatic glioma is a rare clinical entity that is poorly studied in the currently available literature. To contribute, we present a case of GBM formation at the site of a former TBI scar. Although we cannot rule out the possibility that patient had a preexisting tumor during the initial traumatic accident, the likelihood of such scenario is extremely low as the patient achieved a full recovery and remained well for four years following the injury. This is especially true when we take into account a diagnosis of GBM, IDH-wildtype - the most aggressive form of brain cancer with a prognosis of months without any treatment. Even though the majority of large-scale epidemiological studies deny the relationship between TBI and cancer, they ignored the frequency and severity of traumatic insults, certainly important factors in gliomagenesis. The rationale of pathophysiological response following TBI supports such association; therefore, risk between trauma and tumor cannot be ruled out completely and should be studied further in a well-designed and controlled fashion.
